# Reduced gut microbiota-derived indole-3-carboxaldehyde and 5-hydroxyindole-3-acetic acid are associated with intestinal barrier dysfunction and inflammation in diarrheic suckling lambs

**DOI:** 10.1186/s40104-026-01493-w

**Published:** 2026-09-04

**Authors:** Jiahao Li, Zhengli Wang, Xiaoxue Ma, Cunxi Nie, Tingting Liu, Yuanyuan Li, Wenju Zhang

**Affiliations:** 1 College of Animal Science and Technology, Shihezi University, Shihezi, 832000 China; 2 Bingtuan Key Laboratory for Efficient Utilization of Non-Grain Feed Resources, Shihezi University, Shihezi, 832000 China

**Keywords:** 5-Hydroxyindole-3-acetic acid, Gut microbiota, Indole-3-carboxaldehyde, Intestinal barrier, Suckling lambs

## Abstract

**Background:**

Diarrhea in suckling lambs is associated with gut microbiota dysbiosis, impaired intestinal barrier function, and enhanced inflammatory responses. However, the specific intestinal microbes and microbial metabolites linked to intestinal homeostasis in suckling lambs remain unclear.

**Results:**

In this study, diarrheic lambs showed significantly higher serum diamine oxidase (DAO) activity, D-lactate (D-LA), and pro-inflammatory cytokine levels than healthy lambs. We then compared gut microbial composition and metabolite profiles between healthy and diarrheic suckling lambs. Diarrheal lambs exhibited gut microbiota dysbiosis, characterized by an elevated Bacillota (syn. Firmicutes)/Bacteroidota (syn. Bacteroidetes) ratio, reduced abundance of beneficial commensals including *Phocaeicola vulgatus* and *Bacteroides fragilis*, and proliferation of the opportunistic pathogen *Clostridium perfringens*. Metabolomic analysis showed that, in diarrheic lambs, the tryptophan metabolism pathway was reduced with lower levels of indole-3-carboxaldehyde (IAld) and 5-hydroxyindole-3-acetic acid (5-HIAA) than in healthy lambs. Fecal microbiota transplantation experiments showed that transplantation of microbiota from diarrheic lambs partially recapitulated the donor-associated microbial, metabolic, and inflammatory phenotypes in recipient mice. Finally, functional validation using a dextran sulfate sodium (DSS)-induced colitis model revealed that supplementation with IAld and 5-HIAA significantly alleviated DSS-induced intestinal inflammation and barrier damage, accompanied by downregulated expression of genes related to the Tlr4-Myd88-Nfκb signaling pathway.

**Conclusions:**

Our findings indicate that the gut microbiota-derived tryptophan metabolites IAld and 5-HIAA alleviated inflammation and improved intestinal epithelial barrier function by enhancing tight junction integrity, while also reducing the expression of Tlr4/Myd88/Nfκb pathway-related inflammatory signaling molecules.

**Supplementary Information:**

The online version contains supplementary material available at https://doi.org/10.1186/s40104-026-01493-w.

## Introduction

In ruminants, the suckling period is a critical window for gastrointestinal development and the establishment of the immune system [1]. During this stage, the intestinal barrier structure and function of lambs are not yet fully developed, mucosal immune capacity remains relatively immature, and the gut microbiota undergoes rapid colonization and dynamic remodeling. These developmental features result in an increased susceptibility in suckling lambs to diarrhea [2–4]. Once diarrhea occurs, it is commonly accompanied by reduced feed intake, impaired nutrient absorption, gut microbial dysbiosis, and growth retardation, causing substantial losses in lamb production [5, 6]. With the comprehensive prohibition of antibiotics in animal feed, the development of safe and effective antibiotic replacement strategies has emerged as a critical and urgent challenge for the livestock industry [7, 8].

The establishment of intestinal barrier function is a pivotal event in intestinal development during the suckling period [9, 10]. Previous studies have shown that early diarrhea is closely associated with intestinal mucosal damage, reduced goblet cell abundance, impaired mucosal defense, and decreased expression of tight junction proteins [2, 11]. The disruption of intestinal epithelial barrier integrity leads to increased intestinal permeability, facilitating the translocation of bacteria and their components, such as lipopolysaccharide (LPS), across the epithelial barrier into the mucosal layer, thereby initiating or exacerbating inflammatory responses [12].

The gut microbiota is a key regulator of barrier maturation and immune homeostasis [13, 14]. During the suckling period, lambs possess a complex, dynamically evolving gut microbiota that participates in nutrient metabolism and influences host health by regulating intestinal epithelial turnover, mucosal immunity, and inflammatory responses [15]. Studies have shown that diarrhea is associated with decreased gut microbial diversity, reduced beneficial bacteria, expanded opportunistic pathogens, impaired intestinal barrier integrity, and heightened inflammatory responses [16, 17]. A growing body of evidence has indicated that the gut microbiota participates in nutrient digestion and absorption and metabolizes dietary components to produce bioactive molecules. These molecules act as key signals in host-microbe interactions, influencing intestinal barrier integrity, immune homeostasis, and inflammatory responses [18, 19]. However, identifying which specific intestinal microbes and the corresponding metabolites that play critical roles in the anti-diarrheal response of suckling lambs remains challenging.

In recent years, tryptophan metabolism and microbiota-derived indole derivatives have attracted increasing attention because of their role in regulating intestinal epithelial barrier integrity and mucosal immune homeostasis [20]. Gut microbes can metabolize tryptophan into multiple bioactive derivatives, including indole-3-carboxaldehyde (IAld) and 5-hydroxyindole-3-acetic acid (5-HIAA). Previous studies have shown that 5-HIAA improves intestinal barrier function and suppresses inflammatory responses through activation of the AhR pathway, thereby alleviating diarrhea in piglets [21]. IAld reportedly attenuates intestinal epithelial barrier injury and inflammatory responses in colitis or ulcerative colitis models, potentially involving AhR-related and inflammatory signaling pathways [22, 23]. However, although previous studies have suggested that IAld and 5-HIAA are closely associated with intestinal barrier protection and inflammatory regulation, it remains unclear whether these metabolites are altered in the context of diarrhea in suckling lambs, and how they are related to gut microbiota dysbiosis, increased intestinal permeability, and inflammatory responses.

Therefore, this study first compared the gut microbial composition and metabolomic profiles of healthy and diarrheic suckling lambs, and evaluated intestinal barrier permeability and inflammatory status by measuring diamine oxidase (DAO) activity, D-lactate (D-LA) levels, and inflammatory cytokines. Second, a recipient mouse model was established using fecal microbiota transplantation (FMT) to assess whether diarrhea-associated microbiota could transfer related metabolic phenotypes and reproduce barrier and inflammation-related alterations. Finally, IAld and 5-HIAA were functionally evaluated in a dextran sulfate sodium (DSS)-induced colitis model, with a focus on barrier- and inflammation-related responses. We hypothesized that in suckling lambs, diarrhea is associated with gut microbial dysbiosis and altered tryptophan metabolism, accompanied by reduced levels of specific indole metabolites; these changes may be related to impaired intestinal barrier function and enhanced inflammatory responses. This study provides preliminary evidence for a potential microbiota–tryptophan metabolism–intestinal health axis in diarrhea of suckling lambs and may help identify candidate targets for future non-antibiotic intervention strategies.

## Materials and methods

This experiment was conducted at the Fine-wool Sheep Breeding Base of Gongnaisi Sheep Farm, Ili Kazakh Autonomous Prefecture (Xinjiang, China). The experimental ewes were multiparous Xinjiang fine-wool sheep with similar lambing dates. After birth, each lamb was ear-tagged, and maternal information and sex were recorded. All lambs were housed indoors and reared under a feeding system combining suckling and supplementary feeding. From 7 days of age, lambs were provided with pelleted starter feed, with ad libitum access to water and salt bricks. The ewes were in good body condition and produced sufficient milk. The immunization program followed standardized local procedures. No antibiotics or anti-diarrheal drugs were administered to the lambs before sample collection. After sample collection, diarrheic lambs were treated and cared for according to the routine veterinary management procedures of the sheep farm.

A total of 50 newborn male lambs were selected to record the occurrence of diarrhea. During the experimental period, fecal status was assessed twice daily in the lamb pens at 11:00 and 17:00. Diarrhea was scored according to the method described by Marquardt et al. [24] as follows: formed, tubular, or pellet-like feces (A, 0 points); soft but formed feces (B, 1 point); pasty, unformed feces without separation of feces and water (C, 2 points); and liquid, unformed feces with separation of feces and water (D, 3 points). Lambs with diarrhea lasting for more than 2 consecutive days were assigned to the diarrhea group (fecal score ≥ 2). Age-matched lambs that never developed diarrhea were assigned to the healthy group (fecal score < 2). Fresh fecal samples from diarrheic lambs underwent preliminary screening on site using a commercial five-item rapid antigen test kit for diarrhea-associated pathogens (Aijin, Hangzhou, China). The tested pathogens included coronavirus, rotavirus, *Clostridium perfringens*, *Escherichia coli* K99, and *Cryptosporidium*. Lambs with positive rapid antigen results for *E.* *coli* K99 or *C.* *perfringens* were included as candidates for the diarrheic group. Ultimately, 12 healthy lambs and 12 diarrheic lambs were selected for subsequent analyses. The clinical information of the lambs used in this study is provided in Table S1. Fecal samples were collected into sterile, nuclease-free 2-mL cryovials, snap-frozen in liquid nitrogen, and stored at −80 °C for subsequent gut microbiota and untargeted metabolomics analyses. Concurrently, 5 mL of blood was collected from the jugular vein into anticoagulant-free tubes and centrifuged at 3,500 r/min for 15 min. The supernatant was then transferred into 2-mL cryovials and stored at −20 °C for subsequent physiological and biochemical analyses.

### Serum antioxidant index analysis

Serum glutathione peroxidase (GSH-Px) activity, superoxide dismutase (SOD) activity, total antioxidant capacity (T-AOC), and malondialdehyde (MDA) content were measured using commercially available assay kits (Nanjing Jiancheng Bioengineering Institute, Nanjing, China). The catalog numbers and their corresponding assay methods were as follows: A005-1-2, colorimetric method, for GSH-Px activity; A001-1-2, hydroxylamine method, for SOD activity; A015-1-2, colorimetric method, for T-AOC; and A003-1-2, thiobarbituric acid (TBA) method, for MDA content.

### Serum inflammatory cytokines, immune parameters, and intestinal permeability index analysis

Serum inflammatory cytokines, including tumor necrosis factor-α (TNF-α), interleukin-1β (IL-1β), interleukin-10 (IL-10), interleukin-6 (IL-6), and interleukin-22 (IL-22); immune indices, including immunoglobulin G (IgG), immunoglobulin A (IgA), and immunoglobulin M (IgM); and intestinal permeability-related markers, including DAO activity, endotoxin (LPS) level, D-lactate (D-LA), and reactive oxygen species (ROS), were measured using enzyme-linked immunosorbent assay (ELISA) kits (Jingmei Biotechnology Co., Ltd., Jiangsu, China) according to the manufacturer’s instructions.

### DNA extraction and 16S rRNA sequencing analysis

Total microbial DNA was extracted from fecal samples using a commercial DNA extraction kit (Omega Bio-tek, Norcross, GA, USA) according to the manufacturer’s instructions. DNA quality was assessed by 1% agarose gel electrophoresis, and DNA concentration and purity were determined using a NanoDrop 2000 spectrophotometer (Thermo Fisher Scientific, USA). The full-length 16S rRNA gene was amplified by PCR using barcoded primers 27 F (5′-AGRGTTYGATYMTGGCTCAG-3′) and 1492R (5′-RGYTACCTTGTTACGACTT-3′) [25]. Amplicons were verified by 2% agarose gel electrophoresis, quantified with magnetic beads using a Qubit 4.0 fluorometer (Thermo Fisher Scientific, USA). Purified amplicons were pooled at appropriate ratios according to the required sequencing output and used for SMRTbell library preparation with the SMRTbell Prep Kit 3.0, including DNA damage repair, end repair, and adapter ligation. Sequencing was performed on the PacBio Sequel IIe platform by Shanghai Majorbio Bio-Pharm Technology Co., Ltd.

Sequencing reads were demultiplexed according to their barcodes. After length filtering (retaining sequences of 1,000–1,800 bp) and orientation correction, operational taxonomic units (OTUs) were clustered at 97% sequence similarity, and chimeras were removed using UPARSE 7.1 [26, 27]. To minimize the effect of uneven sequencing depth on alpha- and beta-diversity analyses, all samples were rarefied to 6,000 reads per sample (mean Good’s coverage after rarefaction: 99.09%). Taxonomic annotation of OTUs was performed using the RDP classifier (version 2.11) [28] against the SILVA 16S rRNA database (v138) with a confidence threshold of 70%. Community composition at each taxonomic level was then summarized. Functional profiles were predicted using PICRUSt2 (version 2.2.0) [29]. Alpha-diversity indices, including Chao1 and Shannon, were calculated using the mothur software platform [30], and between-group differences were analyzed using Student’s *t*-test. Principal coordinates analysis (PCoA) was performed based on Bray–Curtis distances, and differences in microbial community structure between groups were assessed using PERMANOVA. Linear discriminant analysis effect size (LEfSe) [31] was used to identify differentially abundant taxa from the phylum to genus levels (LDA > 2, *P* < 0.05).

### Untargeted metabolomics analysis

Approximately 20 mg of each fecal sample was weighed into a 2-mL centrifuge tube, and 400 μL of extraction solvent (methanol:water, 4:1, v/v) containing 0.02 mg/mL L-2-chlorophenylalanine as the internal standard was added for metabolite extraction. Samples were homogenized in a cryogenic tissue grinder for 6 min at −10 °C and 50 Hz, followed by ultrasonic extraction at 5 °C for 30 min at 40 kHz. After incubation at −20 °C for 30 min, the extracts were centrifuged at 13,000 × *g* for 15 min at 4 °C. The supernatants were collected and analyzed by LC–MS using a UHPLC-Exploris 240 system at Shanghai Majorbio Bio-Pharm Technology Co., Ltd. Raw LC–MS data were imported into Progenesis QI (Waters Corporation, Milford, USA) for normalization, generating a data matrix containing retention time, mass-to-charge ratio (*m/z*), and peak intensity. Metabolites were identified by matching against the Human Metabolome Database (HMDB), METLIN, and Majorbio’s in-house database. The annotated data matrix was then uploaded to the Majorbio Cloud platform for downstream analyses. Principal component analysis (PCA) and orthogonal partial least squares discriminant analysis (OPLS-DA) were performed on the preprocessed data matrix using *the ropls package* in R (version 1.6.2). Seven-fold cross-validation was used to evaluate model stability. Significantly differential metabolites were identified based on variable importance in projection (VIP) scores from the OPLS-DA model and *P* values from Student’s *t*-test, using thresholds of VIP > 1 and *P* < 0.05. Differential metabolites were annotated to metabolic pathways using the KEGG database. Pathway enrichment analysis was performed using the Python package *scipy.stats*, and Fisher’s exact test was used to identify biological pathways most closely associated with the experimental treatments.

### Fecal microbiota transplantation in mice and sample collection

Approximately 6 g of feces was weighed into a clean beaker, mixed with sterile normal saline at a ratio of 1:5 (w/v), and homogenized with a magnetic stirrer at room temperature for 20 min. The mixture was filtered twice through a sterile 0.25-mm mesh to remove particulate matter. The filtrate was collected, transferred to sterile centrifuge tubes, and centrifuged at 6,000 × *g* for 15 min at 4 °C. After the supernatant was decanted, the pellet was resuspended in an equal volume of sterile physiological saline to obtain the fecal bacterial suspension. The fecal bacterial suspension was thoroughly mixed, glycerol was added to make it up to a final concentration of 10%, and the suspension was stored at −80 °C [17, 32]. One hour before transplantation, the stored suspension was thawed and reactivated in a 37 °C water bath. The fecal bacterial suspension was then immediately administered to mice by oral gavage using a sterile syringe. The viable bacterial count in the fecal bacterial suspension was determined by the plate-counting method to ensure that each preparation contained ≥ 1 × 10^9^ CFU/mL [33].

A total of 18 5-week-old male SPF-grade C57BL/6J mice (20.81 ± 0.37 g; purchased from Henan Skbais Biotechnology Co., Ltd.) were used in this study. Mice were housed under standard conditions (temperature, 25 ± 2 °C; humidity, 55% ± 10%; 12 h light/12 h dark cycle) with ad libitum access to feed and water. After a 7-day acclimation period, the mice were randomly assigned to three groups (*n* = 6 per group): the CON group, which received sterile physiological saline by oral gavage; the FMT_H group, which received a fecal microbiota suspension from healthy lambs; and the FMT_D group, which received a fecal microbiota suspension from diarrheic lambs. Gavage volume was 200 μL per mouse per day. For 7 d prior to fecal microbiota transplantation, all mice received 200 μL/d of a broad-spectrum antibiotic cocktail (ampicillin, 1 g/L; neomycin sulfate, 1 g/L; metronidazole, 1 g/L; vancomycin, 0.5 g/L) by oral gavage to deplete the endogenous gut microbiota. Fecal microbiota transplantation was then performed for 7 consecutive days. At the end of the experiment, blood was collected by ocular enucleation. Serum was then isolated and stored at −20 °C for subsequent physiological and biochemical analyses. The mice were subsequently anesthetized and euthanized. Colonic tissues were collected and rinsed with phosphate-buffered saline (PBS) to remove luminal contents. The tissues were then fixed in 4% paraformaldehyde for 48 h for histomorphological analysis. Fecal samples were also collected, snap-frozen in liquid nitrogen, and stored at −80 °C for subsequent gut microbiota and untargeted metabolomics analyses.

### Mouse indole-3-carboxaldehyde and 5-hydroxyindole-3-acetic acid treatment and sample collection

DSS (36–50 kDa; > 98% purity) was purchased from Beijing Coolaber Science & Technology Co., Ltd. (China). Dimethyl sulfoxide (DMSO; ≥ 99.9% purity) was purchased from Aladdin (Shanghai, China).

A total of 32 5-week-old male SPF-grade C57BL/6J mice (20.31 ± 0.84 g) were used in this study. After a 7-day acclimation period, the mice were randomly assigned to four groups (*n* = 8 per group): CON, DSS, IAld + DSS, and 5-HIAA + DSS. From d 1 to 14 of the experiment, mice in the CON and DSS groups received physiological saline containing 5% DMSO by oral gavage once daily [34]. Mice in the IAld + DSS and 5-HIAA + DSS groups received IAld or 5-HIAA (Aladdin, China) by oral gavage at a dose of 20 mg/kg/d [23, 35]. After the pretreatment period, all mice except those in the CON group received 3% DSS for 7 consecutive days to induce colitis. At the end of the experiment, blood was collected by ocular enucleation. Serum was then isolated and stored at −20 °C for subsequent physiological and biochemical analyses. The mice were subsequently anesthetized and euthanized. Colon length was measured, and colon tissues were collected. One portion was rinsed with PBS to remove luminal contents and fixed in 4% paraformaldehyde for 48 h for histomorphological analysis, whereas the remaining portion was placed into sterile, nuclease-free 2-mL cryovials, snap-frozen in liquid nitrogen, and stored at −80 °C for subsequent qRT-PCR analysis of gene expression.

### Body weight measurement and disease activity index (DAI) assessment

During the experiment, the body weight of each mouse was measured and recorded daily before gavage. DAI was scored based on changes in body weight, stool consistency, and fecal blood, as shown in Table S2. DAI was calculated as (body weight loss score + stool consistency score + fecal blood score)/3.

### Histomorphological analysis of colon tissue

Colon tissue sectioning and histomorphological analysis were performed using hematoxylin and eosin (H&E) staining, as described by Hu et al. [36]. Briefly, colon tissues were fixed in 4% paraformaldehyde for 48 h, dehydrated through a graded ethanol series, embedded in paraffin, sectioned, deparaffinized, and stained with H&E. The stained sections were examined under a light microscope to evaluate colonic histomorphology among the groups.

### RNA preparation and quantitative real-time PCR analysis

Total RNA was extracted from mouse colon tissue using the Foregene Animal Total RNA Isolation Kit (RE-03011; Foregene, Chengdu, China) according to the manufacturer’s instructions. RNA concentration and purity were measured using 1 μL of each sample with a NanoDrop 2000 spectrophotometer (Thermo Fisher Scientific, Waltham, MA, USA). Reverse transcription was performed using a cDNA synthesis kit (RT-01021; Foregene, Chengdu, China) with a PR-96E thermocycler (MIULAB, Hangzhou, China), following the manufacturer’s protocol. Quantitative real-time PCR was performed using the Real Time PCR Easy™-SYBR Green I kit (QP-01012; Foregene, Chengdu, China) on a LineGene 9600 Plus real-time PCR detection system (FQD-96A; BIOER, Hangzhou, China). The target genes included intestinal barrier-related genes, namely claudin-1 (*Cldn1*), tight junction protein 1 (*Tjp1*), and occludin (*Ocln*), and inflammation-related genes, namely *Nfκb*, *Tlr4*, and *Myd88*. The qRT-PCR amplification was performed using a two-step protocol according to the manufacturer’s instructions. Briefly, the cycling conditions were as follows: initial denaturation at 95 °C for 3 min, followed by 40 cycles of denaturation at 95 °C for 10 s and annealing/extension at 60 °C for 20 s. A melting curve analysis was performed after amplification to verify the specificity of the PCR products. Relative mRNA expression levels were calculated using the 2^−ΔΔCt^ method, with β-actin as the internal reference gene [37]. Primer sequences were synthesized by Zhejiang Youkang Biotechnology Co., Ltd. (Urumqi, China), and are listed in Table S3.

### Statistical analysis

Data were initially organized using Microsoft Excel 2019. Statistical analysis was performed using IBM SPSS Statistics 24 (IBM Corp., Armonk, NY, USA). Comparisons between two independent groups were conducted using Student’s unpaired *t*-test. Comparisons among three or more groups were performed using one-way analysis of variance (ANOVA) followed by Duncan’s multiple range test. Graphs were generated using GraphPad Prism 8.0 (Dotmatics, La Jolla, CA, USA). Data are presented as the mean ± standard error of the mean (SEM). Statistical significance was set at *P* ≤ 0.05. Details of the statistical comparisons and significance annotations are provided in the corresponding table footnotes and figure legends.

## Results

### Diarrheal lambs show elevated markers of intestinal permeability, oxidative stress, and inflammation

**Fig. 1:**
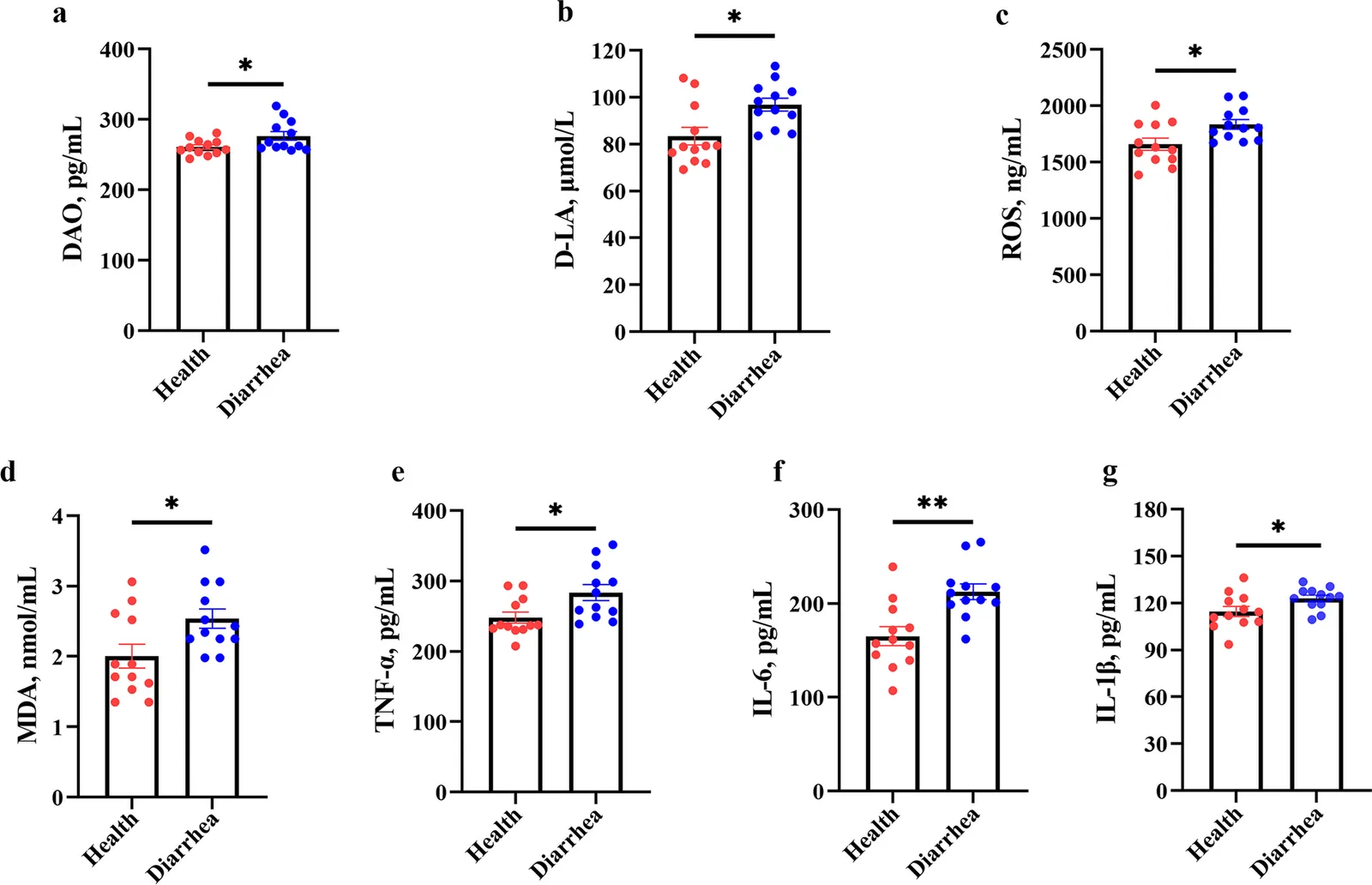
Intestinal barrier function, oxidative stress, and inflammatory response evaluation in healthy and diarrhea suckling lambs. **a** Serum DAO activity. **b** Serum D-LA concentration. **c** Serum ROS concentration. **d** Serum MDA concentration. **e** Serum TNF-α concentration. **f** Serum IL-6 concentration. **g** Serum IL-1β concentration. Data are expressed as the mean ± SEM (*n* = 12). Statistical significance was calculated using Student’s unpaired *t*-test. ^*^P ≤ 0.05, ^**^P ≤ 0.01

To evaluate whether diarrhea was associated with impaired intestinal barrier function, oxidative stress, and systemic inflammation in lambs, serum biomarkers related to intestinal permeability, oxidative status, inflammatory responses, and immunoglobulins were measured. In comparison with healthy lambs, diarrheal lambs showed significantly increased serum DAO and D-LA levels, indicating increased intestinal permeability (*P* < 0.05, Fig. 1a and b). Serum ROS and MDA concentrations were also significantly elevated in diarrheal lambs (*P* < 0.05, Fig. 1c and d), indicative of enhanced oxidative stress. In addition, the pro-inflammatory cytokines IL-6, TNF-α, and IL-1β were significantly increased in diarrheal lambs compared with healthy controls (*P* < 0.05, Fig. 1e–g). Other measured parameters, including LPS, T-AOC, SOD, GSH-Px, IL-10, IgG, IgA, and IgM, did not differ significantly between groups and are summarized in Table S4.

### Diarrheal lambs exhibit gut microbial dysbiosis

**Fig. 2:**
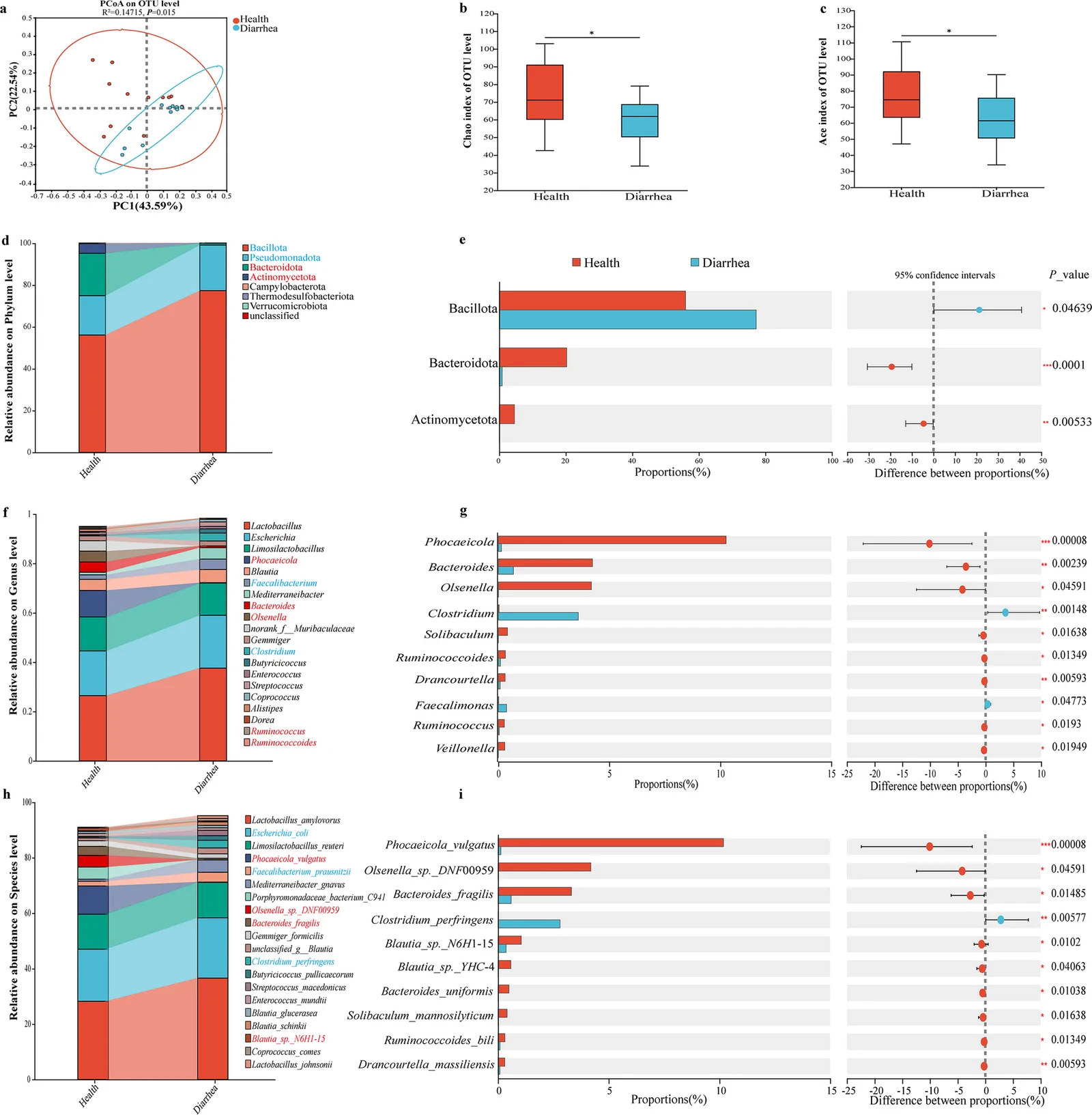
The structure and composition of fecal microbiota in healthy and diarrheal suckling lambs. **a** Principal coordinate analysis (PCoA). **b** Chao index represents the α-diversity. **c** ACE index represents the α-diversity. **d** Phylum-level distribution. **e** Wilcoxon rank-sum test bar plot on phylum level. **f** Genus-level distribution. **g** Wilcoxon rank-sum test bar plot on genus level. **h** Species-level distribution. **i** Wilcoxon rank-sum test bar plot on species level. ^*^P ≤ 0.05, ^**^P ≤ 0.01, ^***^P ≤ 0.001

To investigate differences in the fecal microbiota between healthy and diarrheic lambs, PacBio full-length 16S rRNA gene sequencing was performed to characterize microbial composition and functional potential. Analysis of gut microbial richness and diversity showed that the Chao and ACE indices were significantly higher in healthy lambs than in diarrheic lambs (*P* < 0.05, Fig. 2b and c), whereas the Shannon and Simpson indices did not differ significantly between the groups (*P* > 0.05, Additional file Fig. S1a and b). PCoA showed clear separation between healthy and diarrheic lambs, indicating significant differences in bacterial community composition between the two groups (*R*^2^ = 0.14715, *P* = 0.015, Fig. 2a). At the phylum level, Bacillota, Pseudomonadota, and Bacteroidota were the dominant taxa in both healthy and diarrheic lambs. Compared with healthy lambs, however, diarrheic lambs exhibited a significantly higher relative abundance of Bacillota (*P* < 0.05) and an apparent increasing trend in Pseudomonadota (*P* > 0.05), whereas the relative abundances of Bacteroidota and Actinomycetota were significantly lower (*P* < 0.05, Fig. 2d and e). At the genus level, the relative abundances of *Phocaeicola*, *Bacteroides*, and *Olsenella* were significantly higher in healthy lambs than in diarrheic lambs (*P* < 0.05), whereas *Clostridium* and *Faecalimonas* were enriched in diarrheic lambs (*P* < 0.05, Fig. 2f and g). Notably, these differences in community structure were further evident at the species level. Specifically, several potentially beneficial or commensal species were enriched in healthy lambs, including *Phocaeicola vulgatus*, *Olsenella* sp. DNF00959, *Bacteroides fragilis*, *Blautia* sp. N6H1-15, *Blautia* sp. YHC-4, *Bacteroides uniformis*, *Solibaculum mannosilyticum*, *Ruminococcoides bili*, and *Drancourtella massiliensis*, all of which showed significantly higher relative abundances than in diarrheic lambs (*P* < 0.05). In contrast, the relative abundance of the opportunistic pathogen *Clostridium perfringens* was significantly higher in diarrheic lambs (*P* < 0.05, Fig. 2h and i). These differences in community structure were further supported by LEfSe analysis (Fig. S1c). We further analyzed the core microbiota by classifying taxa according to their prevalence across samples as transient, intermittent, or persistent (Table S5). The proportion of persistent taxa was higher in diarrheic lambs than in healthy lambs (89.52% vs. 80.87%), whereas the proportion of intermittent taxa was lower (9.99% vs. 18.27%). Moreover, persistent microbiota comprised a greater diversity of taxa. These results indicate that the gut microbiota of healthy lambs is enriched in diverse beneficial and commensal bacteria, whereas that of diarrheic lambs exhibited reduced beneficial taxa and increased opportunistic pathogens.

### Radical alteration of metabolite levels

**Fig. 3:**
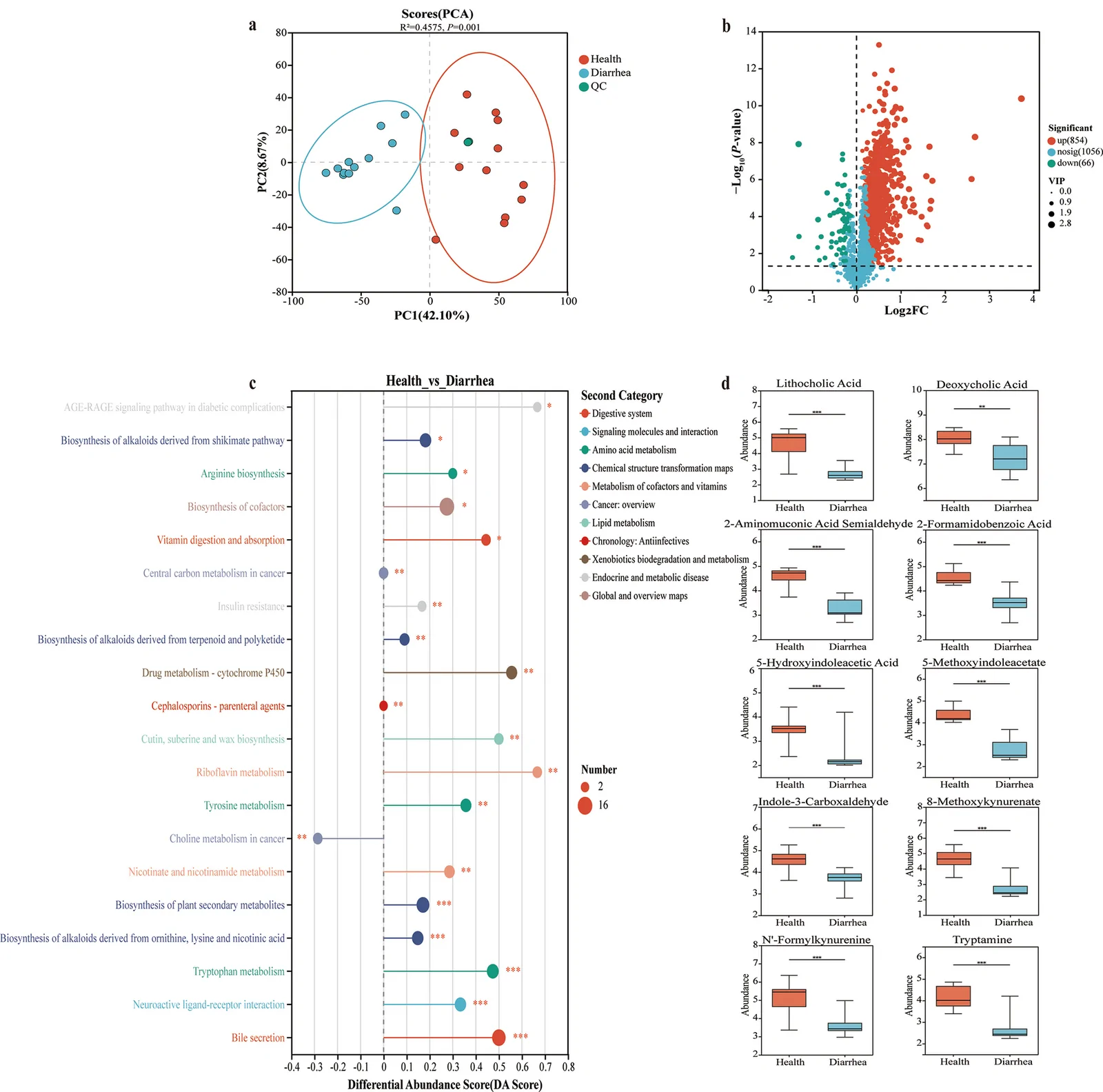
Metabolomic analysis of fecal samples from healthy and diarrhea-affected suckling lambs.** a** Principal component analysis (PCA). **b** Volcano plot of differentially expressed genes. **c** KEGG enrichment map of differentially expressed genes in healthy and diarrheic lambs. **d** Relative abundance of metabolites involved in bile secretion and tryptophan metabolism in healthy and diarrhea lambs. ^*^P ≤ 0.05, ^**^P ≤ 0.01, ^***^P ≤ 0.001

To evaluate the impact of gut microbiota alterations on the intestinal environment, we performed untargeted metabolomic profiling of fecal samples from healthy and diarrheic lambs using UPLC-MS/MS. PCA showed a clear trend of separation in the fecal metabolite profiles between healthy and diarrheic lambs (Fig. 3a). PLS-DA further confirmed that this separation was statistically significant (*R*^2^ = 0.4575, *P* = 0.001), indicative of marked remodeling of the fecal metabolome under diarrheic conditions (Fig. S2a). This shift in metabolic phenotype was highly consistent with the gut microbial community changes revealed by PCoA, suggesting a close association between microbiota alterations and host metabolic phenotype. Venn analysis showed that the healthy and diarrheic groups contained 539 and 76 unique metabolites, respectively, with 1,720 metabolites shared between the groups (Fig. S2b). The volcano plot further revealed marked differences in metabolite abundance between the groups: compared with the diarrheic group, 854 metabolites were upregulated, and 66 were downregulated in the healthy group (Fig. 3b). These differences in metabolite composition could substantially affect the host’s physiological status and function [38]. Chemical classification of the differential metabolites indicated that amino acids, monosaccharides, and carboxylic acids were the major altered components, with amino acids accounting for the largest proportion (16.54%) (Fig. S2c). To further elucidate the biological functions of the differential metabolites, we performed KEGG pathway enrichment analysis. The results showed that these metabolites were mainly enriched in key pathways, including bile secretion, neuroactive ligand-receptor interaction, and tryptophan metabolism (Fig. 3c). Notably, bile secretion and tryptophan metabolism were identified as core metabolic pathways closely associated with diarrhea [39, 40]. Specifically, within the bile secretion pathway, the levels of deoxycholic acid, lithocholic acid, and hyodeoxycholic acid were significantly higher in the healthy group than in the diarrheic group. Similarly, in the tryptophan metabolism pathway, metabolites such as indole-3-carboxaldehyde, 5-hydroxyindoleacetic acid, 5-methoxyindoleacetate, tryptamine, and 8-methoxykynurenate showed the same trend, suggesting that they may play key roles in maintaining gut health (Fig. 3d).

**Fig. 4:**
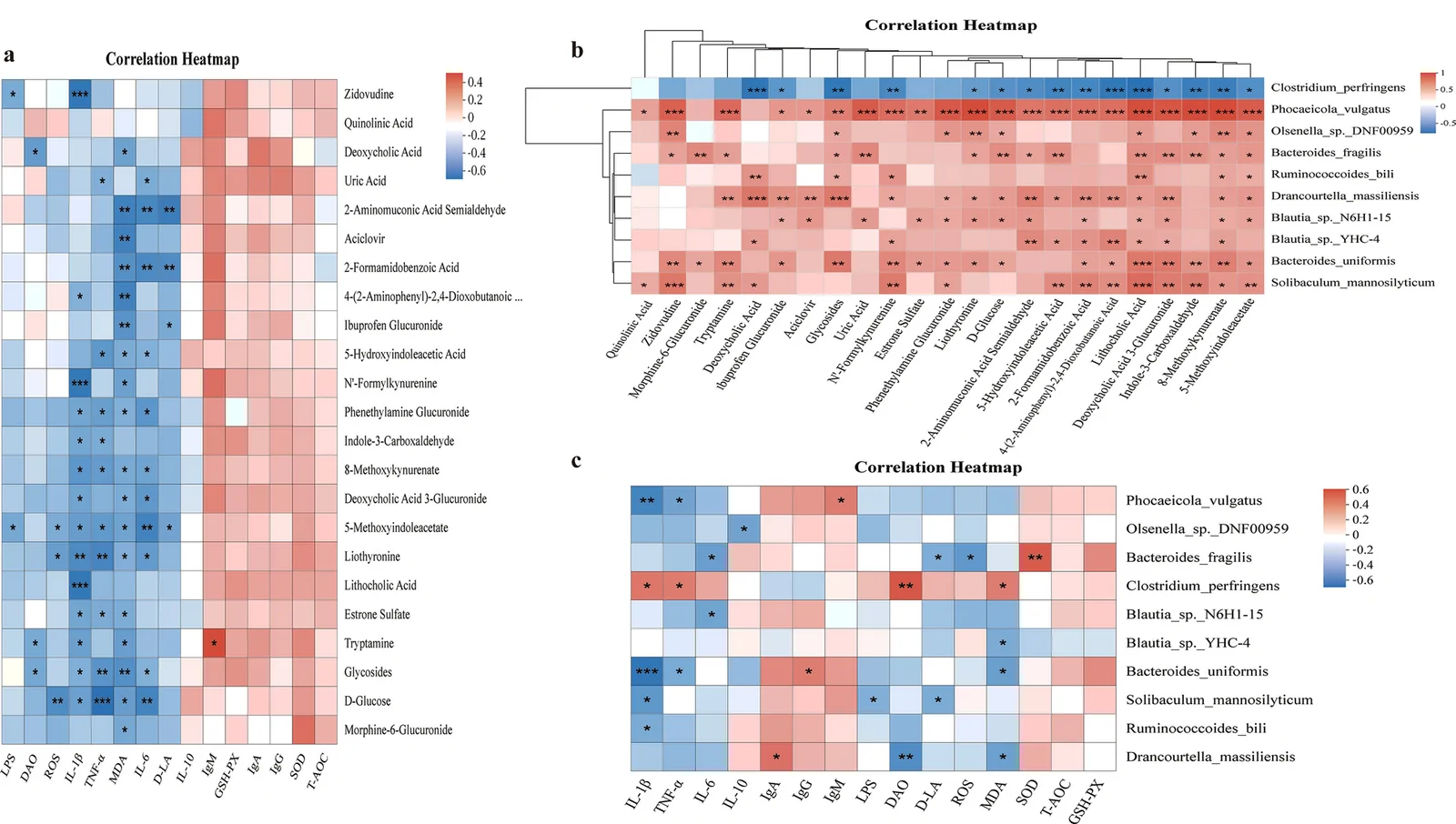
Spearman correlations among differential metabolites, physiological/biochemical indicators, and gut microbiota in healthy and diarrheic lambs. **a** Correlation analysis of differential metabolites with physiological and biochemical indicators. **b** Correlation analysis of differential metabolites with differential microorganisms. **c** Correlation analysis of differential microorganisms with physiological and biochemical indicators. Red denotes a positive correlation; blue denotes a negative correlation. The color intensity is proportional to the strength of the Spearman correlation. ^*^P ≤ 0.05, ^**^P ≤ 0.01, ^***^P ≤ 0.001

Spearman correlation analysis further revealed associations of differential metabolites with lamb physiological indices and the gut microbiota. The results showed that the metabolites identified from the bile acid and tryptophan metabolism pathways were positively correlated with immunoglobulins (IgA, IgG, and IgM) and antioxidant indicators (SOD, T-AOC, and GSH-Px), but negatively correlated with pro-inflammatory cytokines and intestinal permeability markers (e.g., IL-1β, TNF-α, IL-6, DAO, and D-LA). In addition, these metabolites were significantly positively correlated with the abundances of several beneficial bacteria, including *P. vulgatus*, *Olsenella* sp. DNF00959, *Ruminococcoides bili*, and *B. fragilis*. In contrast, these metabolites were significantly negatively correlated with the potential pathogen *C. perfringens*, which was positively correlated with pro-inflammatory cytokines and intestinal permeability markers (Fig. 4a–c). In summary, diarrhea in lambs was associated with dysregulation of the bile secretion and tryptophan metabolism pathways, characterized by a marked reduction in multiple beneficial metabolites. These alterations may have disrupted gut microbial homeostasis and exacerbated intestinal inflammatory responses.

### FMT alters body weight, intestinal permeability, and inflammatory cytokines in mice

**Fig. 5:**
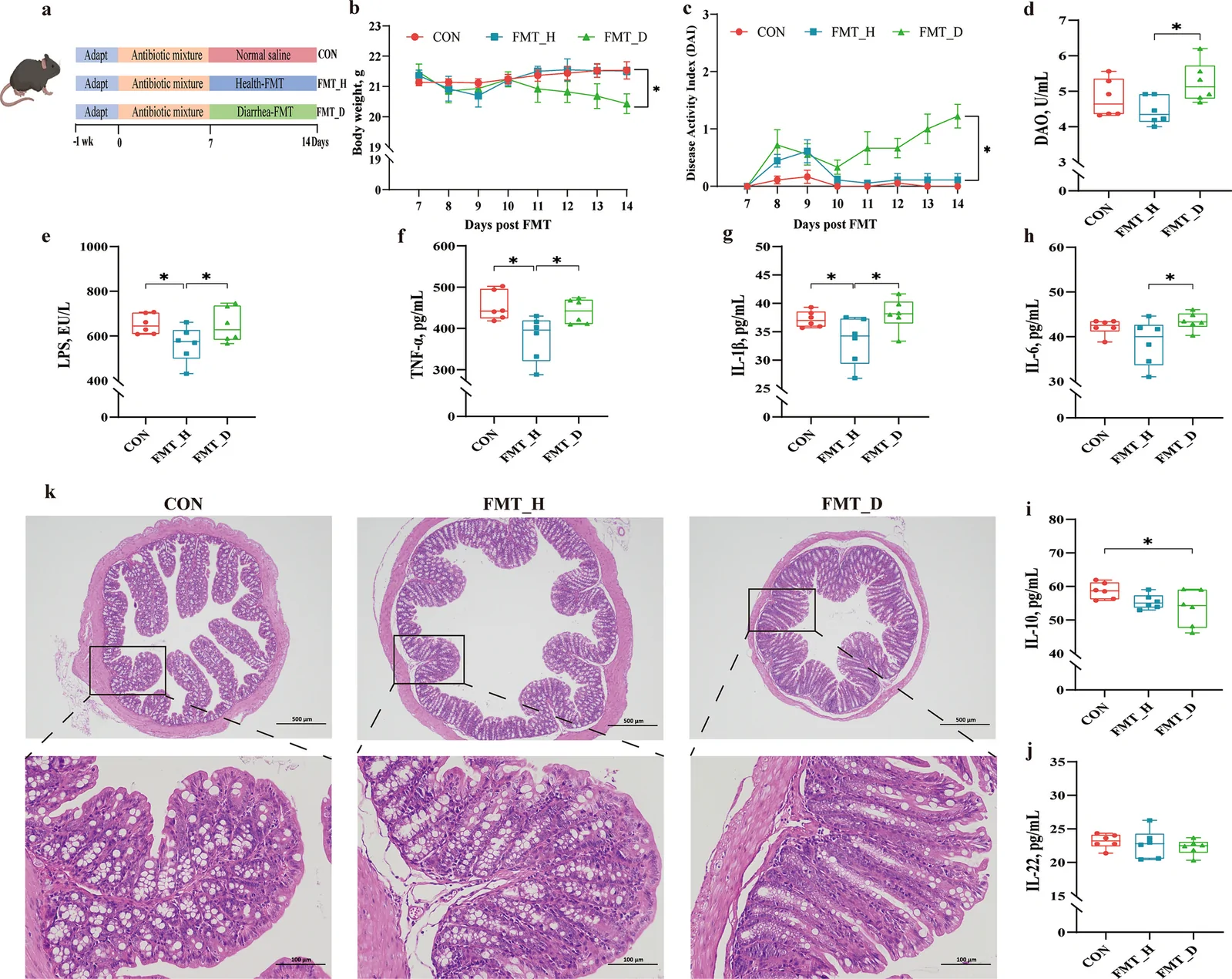
Effects of FMT on body weight, intestinal permeability, and inflammatory cytokines in mice. **a** Experimental design for mouse study. **b** Body weight. **c** Disease activity index. **d** Serum DAO activity. **e** Serum LPS concentration. **f** Serum TNF-α concentration. **g** Serum IL-1β concentration. **h** Serum IL-6 concentration. **i** Serum IL-10 concentration. **j** Serum IL-22 concentration. **k** Representative H&E staining of mouse colon tissue sections (Scale bars, 500 μm and 100 μm). Data are expressed as the mean ± SEM (*n* = 6). Statistical significance was calculated via one-way analysis of variance (ANOVA) followed by Duncan’s multiple range test. ^*^P ≤ 0.05

Fecal microbiota transplantation can potentially restore gut microbial homeostasis by reshaping the composition and structure of the host intestinal microbiota. To evaluate whether microbiota from healthy or diarrheic lambs could transfer donor-associated host-response phenotypes, fecal microbial suspensions were transplanted into antibiotic-pretreated mice (Fig. 5a). During the experiment, body weight and DAI were recorded daily, and fresh fecal samples were collected for gut microbiota profiling. Mice in the FMT_D group exhibited a significant decrease in body weight and a marked increase in DAI (*P* < 0.05, Fig. 5b and c). In terms of intestinal permeability and inflammation, compared with the FMT_H group, the FMT_D group exhibited significantly higher serum levels of DAO, LPS, TNF-α, IL-1β, and IL-6 (*P* < 0.05, Fig. 5d–h). Similarly, the CON group also showed significantly higher levels of LPS, TNF-α, and IL-1β than the FMT_H group (*P* < 0.05, Fig. 5e–g). In addition, serum IL-10 levels were significantly lower in the FMT_D group than in the CON group (*P* < 0.05, Fig. 5i). Histological examination of the colon showed that, compared with the FMT_H group, mice in the FMT_D group exhibited mucosal injury, characterized by reduced mucosal folds, fewer goblet cells, focal lymphocyte aggregation, and uneven muscular layer thickness (Fig. 5k).

### FMT-induced remodeling of the intestinal microbiota in germ free mice

**Fig. 6:**
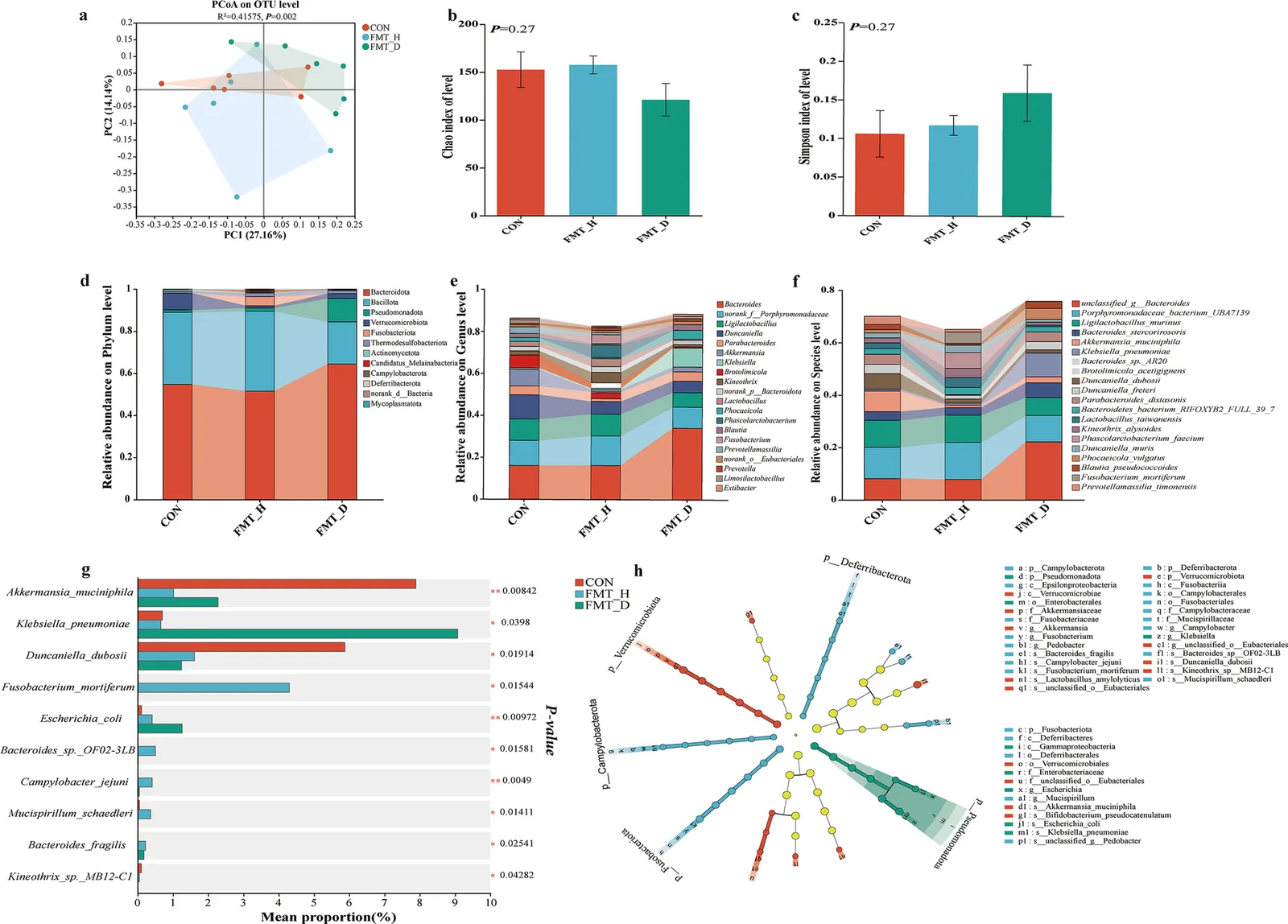
FMT induced remodeling of the intestinal microbiota in germ free mice. **a** Principal coordinate analysis (PCoA). **b** Chao index represents the α-diversity.** c** Simpson index represents the α-diversity.** d** Phylum-level distribution. **e** Genus-level distribution. **f** Species-level distribution. **g** Based on the Kruskal–Wallis rank-sum test for significant differences at the species level. **h** Cladogram. ^*^P ≤ 0.05, ^**^P ≤ 0.01

We analyzed the gut microbiota composition of mice after FMT. Alpha-diversity analysis showed that although the Chao and Simpson indices did not differ significantly among groups (*P* > 0.05, Fig. 6b and c), microbial richness and diversity in the FMT_D group tended to decrease. PCoA showed a significant separation in microbial community structure among the CON, FMT_H, and FMT_D groups (*R*^2^ = 0.41575, *P* = 0.002, Fig. 6a), indicating that FMT had a pronounced effect on gut microbiota structure. To further investigate whether donor microbiota stability could be transmitted, we analyzed the core microbiota structure of recipient mice (Table S5). The proportion of persistent taxa was higher in the FMT_D group (82.20%), similar to the level observed in diarrheic donors and indicating microbiota stability characteristics similar to those of the donors. In contrast, the proportion of persistent taxa in the FMT_H group decreased to 66.19%, whereas the proportion of intermittent taxa increased to 32.81%, suggesting that the microbiota was in a more dynamic transitional state. Taxonomic analysis further supported this trend, indicating that donor microbiota stability characteristics could be transferred via FMT. At the phylum level (Fig. 6d; Fig. S3c), Bacteroidota, Bacillota, Pseudomonadota, Verrucomicrobiota, and Fusobacteriota were the dominant phyla across all groups, and the relative abundance of Pseudomonadota was significantly higher in the FMT_D group (*P* < 0.05). At the genus level (Fig. 6e; Fig. S3d), the FMT_D group was characterized by enrichment of *Klebsiella* and *Escherichia*, whereas *Lactobacillus* was relatively depleted. Species-level analysis identified 10 species that differed significantly among groups, including *Akkermansia muciniphila*, *Klebsiella pneumoniae*, *Fusobacterium mortiferum*, *E. coli*, and *B. fragilis* (Fig. 6f and g). LEfSe further identified group-specific enriched taxa (Fig. S3e): the CON group was mainly characterized by *Bifidobacterium pseudocatenulatum* and *A. muciniphila*; the FMT_H group was predominantly enriched in *Bacteroides* sp. OF02-3LB and *B. fragilis*; whereas the FMT_D group showed significant enrichment of *K. pneumoniae* and *E. coli*. Phylogenetic tree analysis further revealed that the FMT_D group exhibited systematic enrichment of opportunistic pathogens from the phylum to species levels. The higher-level taxa harboring these pathogens, including Gammaproteobacteria, Enterobacterales, and Enterobacteriaceae, were likewise significantly enriched in the FMT_D group (Fig. 6h). These findings were consistent with the microbial features observed in male suckling lambs and collectively highlighted a typical pattern of gut dysbiosis during diarrhea, namely reduced beneficial bacteria and increased pathogenic bacteria.

### Changes in metabolites in mice after FMT

**Fig. 7:**
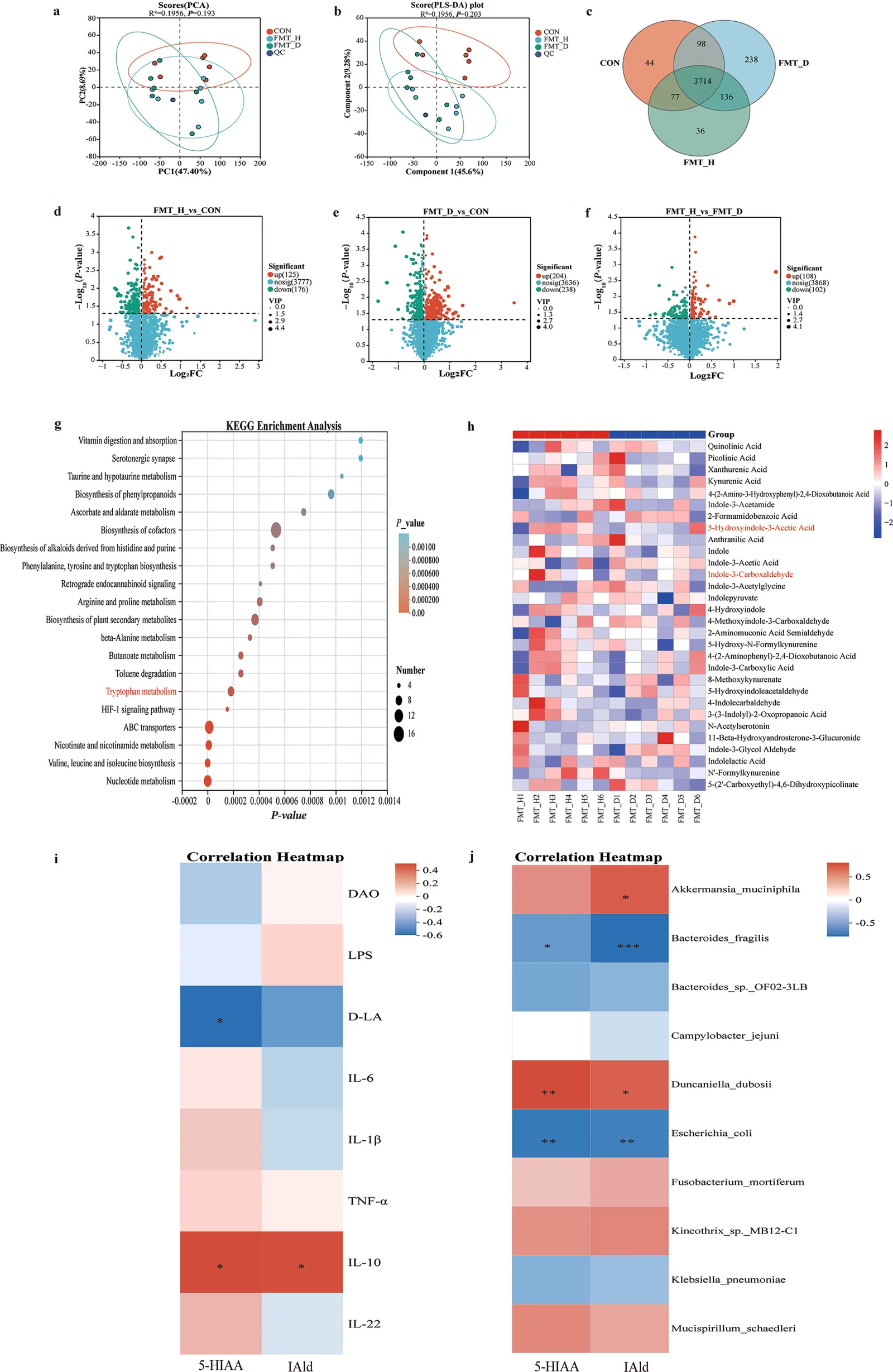
Changes in metabolites in mice after FMT. **a** Principal component analysis (PCA). **b** Partial least squares discriminant analysis (PLS-DA). **c** Venn analysis. **d–f** Volcano plot of differentially expressed genes (FMT_H vs. CON, FMT_D vs. CON, FMT_H vs. FMT_D). **g** KEGG enrichment map of differentially expressed genes. **h** Heatmap visualization of relative abundance of tryptophan metabolites. **i** Correlation analysis of IAld and 5-HIAA with physiological indicators. **j** Correlation analysis of IAld and 5-HIAA with differential microorganisms. Red denotes a positive correlation; blue denotes a negative correlation. ^*^P ≤ 0.05, ^**^P ≤ 0.01, ^***^P ≤ 0.001

To further assess whether donor microbiota transplantation was associated with changes in host metabolic phenotypes, we performed untargeted metabolomic analysis in recipient mice. PCA showed clear separation between the CON group and both the FMT_H and FMT_D groups. A degree of separation was also observed between the FMT_H and FMT_D groups, suggesting differences in metabolite profiles among treatments (Fig. 7a). PLS-DA further supported this separation pattern (*R*^2^ = 0.1956, *P* = 0.203), with the CON group being more distinctly separated from the other groups, and a more evident clustering and separation trend between the FMT_H and FMT_D groups (Fig. 7b). Venn analysis showed that the CON, FMT_H, and FMT_D groups contained 44, 238, and 36 unique differential metabolites, respectively, with 3,714 differential metabolites shared among all three groups (Fig. 7c). Volcano plots further revealed differences in metabolite abundance between groups. For the CON group, the FMT_D group showed 204 upregulated and 238 downregulated metabolites, whereas the FMT_H group showed 125 upregulated and 176 downregulated metabolites. In the key comparison between the FMT_H and FMT_D groups, 108 metabolites were upregulated, and 102 metabolites were downregulated in the FMT_H group (Fig. 7d–f). KEGG pathway enrichment analysis of the differential metabolites revealed significant enrichment of the tryptophan metabolism pathway (Fig. 7g). Notably, this pathway showed high consistency between donor lambs and recipient mice, indicating that the tryptophan metabolism pathway may be a key target of FMT-mediated remodeling. We therefore performed a heatmap analysis of all metabolites in this pathway. The results showed that IAld and 5-HIAA exhibited a consistent upregulation trend in the FMT_H group, and their variation patterns were consistent with those observed in the donor lambs (Fig. 7h). Pearson correlation analysis was then used to assess the relationships of IAld and 5-HIAA with mouse physiological indices and the gut microbiota. The results showed that IAld was negatively correlated with D-LA and IL-6, but positively correlated with IL-10. 5-HIAA was negatively correlated with DAO, LPS, and D-LA, and positively correlated with IL-10 and IL-22. In addition, both IAld and 5-HIAA were negatively correlated with the opportunistic pathogens *K. pneumoniae*, *E. coli*, and *B. fragilis*, but positively correlated with beneficial or commensal bacteria such as *A. muciniphila* and *Duncaniella dubosii* (Fig. 7i and j). In summary, these results identified IAld and 5-HIAA as candidate tryptophan-related metabolites associated with the healthy microbiota-linked phenotype, providing a rationale for their subsequent functional evaluation.

### IAld and 5-HIAA show potential anti-inflammatory effects in DSS-treated mice

**Fig. 8:**
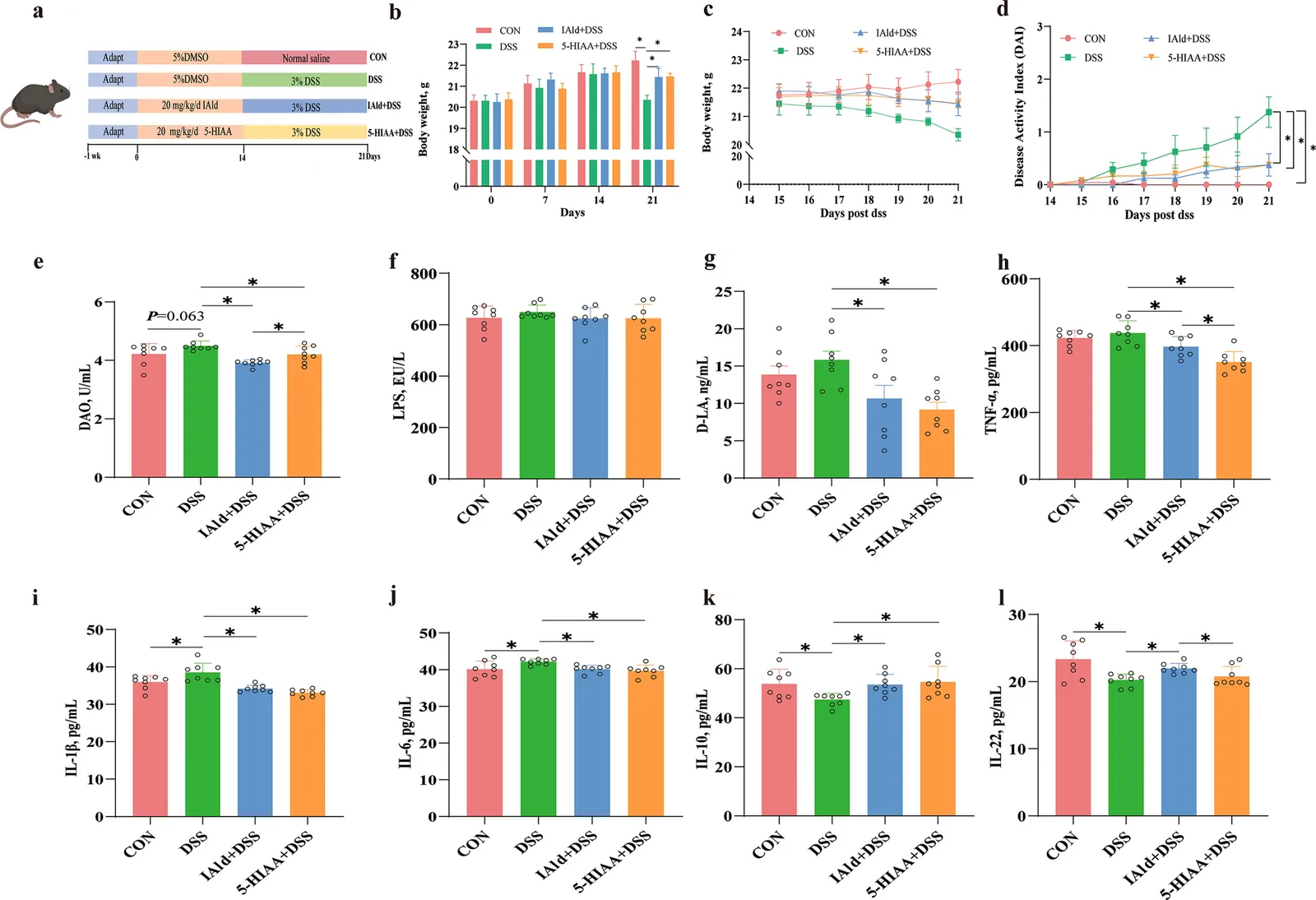
IAld and 5-HIAA alleviate DSS-induced inflammatory responses in mice. **a** Experimental design for mouse study. **b** Body weight. **c** Body weight changes following DSS induction.** d** Disease activity index. **e** Serum DAO activity. **f** Serum LPS concentration. **g** Serum D-LA concentration. **h** Serum TNF-α concentration. **i** Serum IL-1β concentration. **j** Serum IL-6 concentration. **k** Serum IL-10 concentration. **l** Serum IL-22 concentration. Data are expressed as the mean ± SEM (*n* = 8). Statistical significance between the CON and DSS groups was determined using Student’s unpaired *t*-test to assess DSS-induced intestinal injury. Differences among the DSS, IAld + DSS, and 5-HIAA + DSS groups were evaluated using one-way ANOVA followed by Duncan’s multiple range test to assess whether IAld and 5-HIAA pretreatment modulated DSS-induced intestinal injury and inflammatory responses. ^*^P ≤ 0.05

To explore whether IAld and 5-HIAA may influence DSS-induced inflammatory responses, mice were treated with IAld or 5-HIAA at a dose of 20 mg/kg/d by oral gavage for 14 consecutive days, followed by 3% DSS administration for 7 consecutive days (Fig. 8a). During the experiment, body weight and DAI were monitored daily, and colonic injury was assessed. Both IAld and 5-HIAA significantly alleviated DSS-induced body weight loss in mice (*P* < 0.05, Fig. 8b and c) and markedly reduced DAI (*P* < 0.05, Fig. 8d). In terms of intestinal permeability, serum DAO and D-LA levels were significantly higher in the DSS group than in the IAld + DSS and 5-HIAA + DSS groups (*P* < 0.05, Fig. 8e and g). For pro-inflammatory cytokines, serum TNF-α, IL-1β, and IL-6 levels were significantly higher in the DSS group than in the CON, IAld + DSS, and 5-HIAA + DSS groups (*P* < 0.05, Fig. 8h–j). In contrast, serum levels of the anti-inflammatory cytokine IL-10 were significantly higher in the CON, IAld + DSS, and 5-HIAA + DSS groups than in the DSS group (*P* < 0.05, Fig. 8k). In addition, IL-22 levels were significantly higher in the CON and IAld + DSS groups than in the DSS group (*P* < 0.05). IL-22 levels were also significantly higher in the IAld + DSS group than in the 5-HIAA + DSS group (*P* < 0.05, Fig. 8l).

### Analysis of the potential roles of IAld and 5-HIAA in DSS-induced colitis in mice

**Fig. 9:**
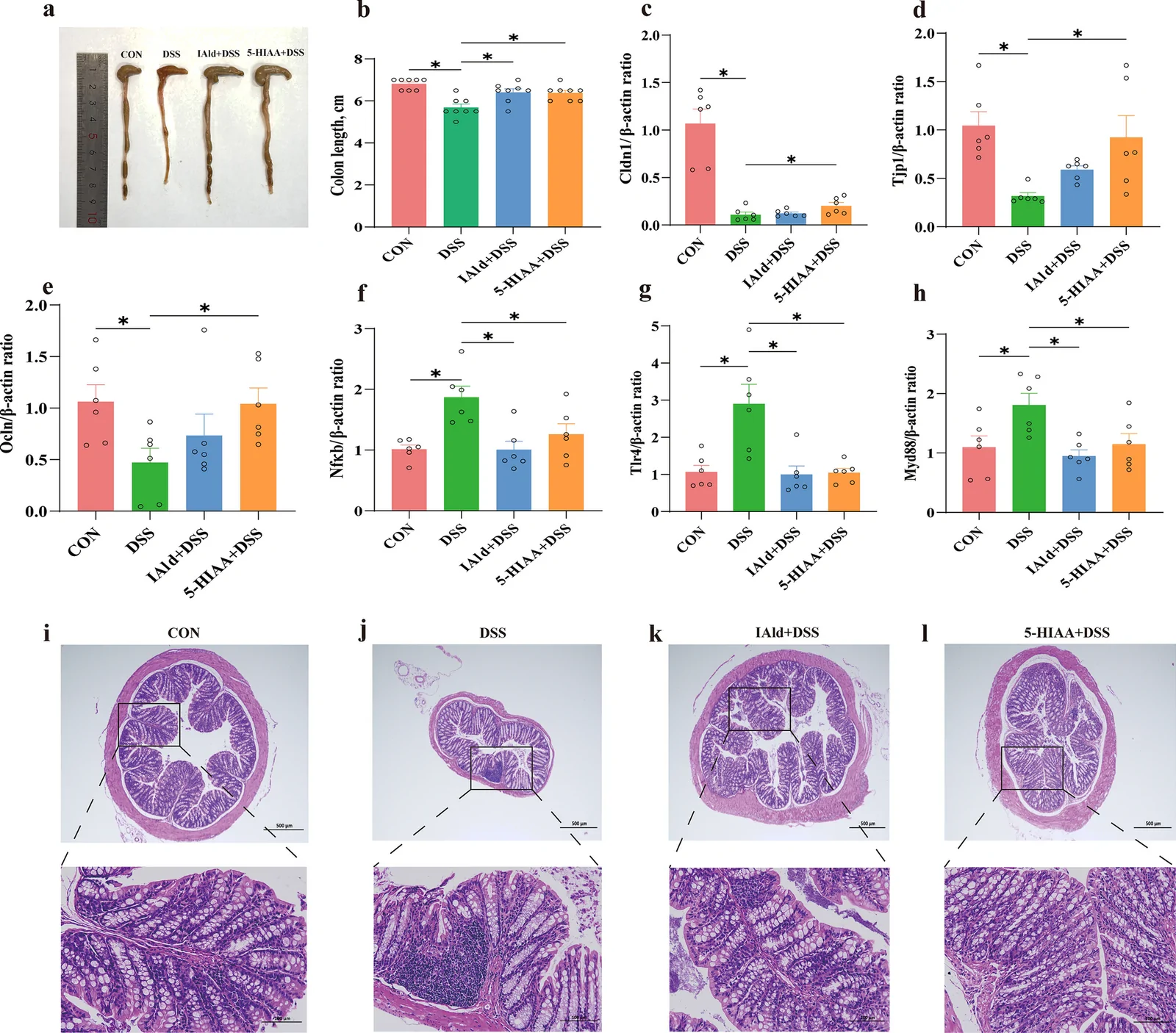
Preventive effect of IAld and 5-HIAA on DSS-induced colitis in mice. **a** Macroscopic appearance. **b** Colon length. **c** Cldn1/β-actin ratio. **d** Tjp1/β-actin ratio. **e** Ocln/β-actin ratio. **f** Nfκb/β-actin ratio.** g** Tlr4/β-actin ratio. **h** Myd88/β-actin ratio. **i–l** Representative H&E staining of mouse colon tissue sections (Scale bars, 500 μm and 100 μm). Data are expressed as the mean ± SEM (*n* = 6). Statistical analysis was performed using the same approach as described for Fig. 8. ^*^P ≤ 0.05

To further explore the potential roles of IAld and 5-HIAA in DSS-induced colitis, we performed H&E staining and qRT-PCR analysis of mouse colonic tissues. The results showed that DSS induction caused pronounced colonic injury in mice, including colon shortening, tissue hyperemia and edema, and ulcer formation (Fig. 9a). Treatment with IAld or 5-HIAA alleviated these pathological lesions and significantly restored colon length (*P* < 0.05, Fig. 9b). Regarding the expression of intestinal barrier-related genes, the DSS group showed significantly reduced mRNA levels of *Cldn1*, *Tjp1*, and *Ocln* compared with the CON group (*P* < 0.05, Fig. 9c–e). Notably, both the IAld + DSS and 5-HIAA + DSS groups showed increased expression of *Cldn1*, *Tjp1*, and *Ocln*, with significant increases observed in the 5-HIAA + DSS group (*P* < 0.05). Moreover, *Tjp1* and *Ocln* expression were restored to levels close to those in the CON group, suggesting that 5-HIAA may be particularly effective in maintaining intestinal barrier integrity. Analysis of inflammatory signaling pathways showed that the mRNA expression levels of *Nfκb*, *Tlr4*, and *Myd88* in colonic tissues were significantly increased in the DSS group (*P* < 0.05, Fig. 9f–h). In contrast, IAld and 5-HIAA treatment effectively suppressed excessive activation of this pathway and reduced the transcriptional levels of these pro-inflammatory signaling molecules (*P* < 0.05). Histological examination of the colon further showed that the DSS group exhibited fewer goblet cells, disorganized crypt architecture, and lymphocyte infiltration and aggregation. In contrast, the IAld + DSS and 5-HIAA + DSS groups showed marked improvement in colonic mucosal morphology compared with the DSS group. Their colonic tissues displayed a more orderly epithelial arrangement, more intact mucosal architecture, and abundant goblet cells, closely resembling the morphology observed in the CON group (Fig. 9i–l). In summary, these results suggest that IAld and 5-HIAA exert protective effects against DSS-induced intestinal injury, accompanied by improved intestinal barrier-related gene expression and reduced mRNA expression of Tlr4/Myd88/Nfκb pathway-related inflammatory signaling molecules.

## Discussion

During the lactation period, lambs undergo rapid development of intestinal structure and function. Their intestinal barrier and microbial community are not yet fully stabilized, making them susceptible to diarrhea, intestinal barrier damage, and dysbiosis [1, 4, 41]. Previous studies have demonstrated that the gut microbiota metabolizes dietary components to produce various bioactive molecules, among which indole derivatives generated from tryptophan metabolism are closely associated with intestinal barrier integrity and mucosal immune regulation [42–44]. Therefore, in the present study, we compared the gut microbiota and its metabolites between healthy and diarrheic lambs and further conducted FMT and metabolite intervention experiments. Our study revealed that diarrheic lambs exhibited altered gut microbiota composition, with decreased levels of tryptophan metabolites IAld and 5-HIAA. Meanwhile, serum levels of DAO, D-LA, and pro-inflammatory cytokines were significantly elevated in diarrheic lambs. FMT experiments showed that transplantation of microbiota from diarrheic lambs partially recapitulated donor-associated microbial, metabolic, and inflammatory phenotypes in recipient mice. Further functional validation in the DSS-induced colitis mouse model indicated that IAld and 5-HIAA supplementation alleviated intestinal inflammation and barrier damage, accompanied by reduced expression of genes related to the Tlr4/Myd88/Nfκb signaling pathway. Collectively, our findings provide preliminary evidence for a potential “microbiota–tryptophan metabolism–intestinal health” axis in lamb diarrhea during the lactation period.

Gut microbiota dysbiosis is often characterized by a reduction in potentially beneficial bacteria and an increase in opportunistic pathogens or pathogenic bacteria [45, 46]. In the present study, PCoA analysis showed a clear separation in gut microbial community structure between healthy and diarrheic lambs. At the species level, the relative abundances of *P. vulgatus*, *B. fragilis*, and *B. uniformis* were significantly decreased in diarrheic lambs, whereas the abundance of *C. perfringens* was markedly increased. These findings are consistent with those reported by Zhong et al. [16]. indicating that diarrheic lambs in the present study exhibited pronounced alterations in gut microbiota composition, mainly characterized by a decrease in several common commensal bacteria and enrichment of the potential pathogen *C. perfringens*. Previous studies have shown that *P. vulgatus* and *B. fragilis* are involved in short-chain fatty acid production and the regulation of intestinal barrier and immune functions [47, 48], whereas *C. perfringens* induces intestinal barrier damage and promotes inflammatory responses [49]. Taken together, these alterations in microbial composition may be associated with the intestinal barrier dysfunction and inflammatory phenotype observed in diarrheic lambs in this study.

The metabolomic results of the present study showed clear differences between healthy and diarrheic lambs in metabolites associated with the bile secretion and tryptophan metabolism pathways. Specifically, the abundances of deoxycholic acid, lithocholic acid, indole-3-carboxaldehyde, 5-hydroxyindoleacetic acid, and 5-methoxyindoleacetate were significantly higher in healthy lambs than in diarrheic lambs. Further correlation analysis showed that these differential metabolites were positively correlated with immunoglobulins (IgA, IgG, and IgM) and antioxidant indices (SOD, T-AOC, and GSH-PX), but negatively correlated with pro-inflammatory cytokines and intestinal permeability markers, including IL-1β, TNF-α, IL-6, DAO, and D-LA. These findings suggest that these metabolites are closely associated with immune status, oxidative stress, and intestinal barrier function in lambs. Previous studies have indicated that certain metabolites derived from the bile secretion and tryptophan metabolism pathways are involved in the regulation of intestinal homeostasis. Kiriyama et al. [50] reported that deoxycholic acid and lithocholic acid can suppress inflammatory responses by activating the TGR5 receptor. Zhang et al. [46] found that IAld promotes intestinal stem cell expansion in piglets and improved intestinal barrier function, whereas Han et al. [21] reported that 5-HIAA enhances intestinal barrier integrity and inhibits inflammation. Taken together, the relative enrichment of these differential metabolites in healthy lambs, together with their significant correlations with immune, antioxidant, and intestinal function-related indicators, suggests that they are closely linked to intestinal health status in lambs.

In the present study, FMT was performed to investigate the effects of diarrhea-associated microbiota on the gut microbial community and metabolic phenotype of recipient mice. The results showed a clear separation in microbial community structure among the CON, FMT_H, and FMT_D groups. Specifically, the CON group was mainly characterized by *B. pseudocatenulatum* and *A. muciniphila*, whereas the FMT_H group was enriched in *Bacteroides* sp. OF02-3LB and *B. fragilis*. In contrast, the FMT_D group was predominantly enriched in *K. pneumoniae* and *E. coli*. These microbial features were consistent with those observed in the donor lambs, suggesting that diarrhea-associated microbiota can successfully colonize the recipient mouse gut and retain their compositional characteristics. Untargeted metabolomic analysis showed that the tryptophan metabolism pathway in FMT_D mice exhibited a trend similar to that observed in diarrheic donor lambs, with decreased levels of IAld and 5-HIAA. Simultaneously, serum levels of DAO, LPS, TNF-α, IL-1β, and IL-6 were significantly elevated in FMT_D mice. Histological observation of colon tissues revealed reduced mucosal folds, decreased goblet cell numbers, focal lymphocyte aggregation, and uneven thickness of the muscular layer in the FMT_D group. Further correlation analysis showed that IAld was negatively correlated with D-LA and IL-6 but positively correlated with IL-10. In addition, 5-HIAA was negatively correlated with DAO, LPS, and D-LA, and significantly positively correlated with IL-10 and IL-22. Collectively, these results indicate that, in the present model, transplantation of microbiota from diarrheic lambs partially recapitulated the donor-associated microbial, metabolic, and inflammatory phenotypes in recipient mice. Notably, the trends in IAld and 5-HIAA levels in recipient mice were consistent with those observed in donor lambs and were significantly correlated with intestinal barrier- and inflammation-related indicators. Therefore, IAld and 5-HIAA were selected as key metabolites for subsequent functional validation.

To further investigate the roles of IAld and 5-HIAA in the regulation of intestinal barrier function and inflammation, in the present study, we established a 3% DSS-induced colitis mouse model to evaluate their protective effects against DSS-induced intestinal injury. Intestinal barrier integrity primarily depends on tight junction proteins, among which ZO-1, Occludin, and Claudin-1 are key components involved in maintaining intercellular epithelial connections. When these structures are disrupted, microbial-associated molecules such as LPS can more readily translocate across the barrier and induce inflammatory responses [51–53]. The results of the present study showed that intervention with either IAld or 5-HIAA alleviated DSS-induced colon shortening. Moreover, both the IAld + DSS and 5-HIAA + DSS groups showed increased gene expression levels of *Cldn1*, *Tjp1*, and *Ocln*. Liu et al. [23] reported that IAld upregulated the expression of *Tjp1* and *Ocln* and reduced the levels of IL-1β, IL-6, and TNF-α, thereby alleviating symptoms of ulcerative colitis in mice. Zhang et al. [46] found that dietary supplementation with IAld improved villus morphology, increased the number of MUC2-positive cells, and enhanced tight junction protein expression in weaned piglets. Zhong et al. [54] reported that 5-HIAA alleviated colonic inflammation by upregulating Occludin and Claudin expression, suppressing pro-inflammatory cytokines, and increasing anti-inflammatory cytokine levels. Consistent with previous findings, the results of the present study suggest that IAld and 5-HIAA interventions are associated with the restoration of tight junction protein expression and improvement of barrier-related indicators. Regarding inflammatory signaling, TLR4 recognizes LPS and activates the MyD88-dependent NF-*κ*B signaling cascade, thereby promoting the production of pro-inflammatory cytokines such as TNF-α, IL-1β, and IL-6 [55–57]. In the present study, the levels of *Nfκb*, *Tlr4*, and *Myd88* were significantly increased in colon tissues from the DSS group, whereas IAld and 5-HIAA interventions reduced the expression levels of *Tlr4*, *Myd88*, and *Nfκb-*related molecules. These findings suggest that the anti-inflammatory effects of IAld and 5-HIAA may be associated with attenuation of this signaling axis. Serum analysis further showed that, compared with the DSS group, the IAld + DSS and 5-HIAA + DSS groups had significantly lower levels of DAO, D-LA, TNF-α, IL-1β, and IL-6, along with significantly higher levels of the anti-inflammatory cytokine IL-10. Notably, IL-22 levels were significantly higher in the IAld + DSS group than in the 5-HIAA + DSS group. Taken in conjunction with previous reports linking IAld to mucosal immune regulation [58], this finding suggests that IAld may promote mucosal repair through IL-22-related pathways; however, the underlying mechanism requires further validation. Overall, the results of this study indicate that, in a DSS-induced mouse colitis model, pretreatment with IAld and 5-HIAA protected against intestinal barrier injury and inflammatory responses; these effects may be associated with increased expression of tight junction proteins and attenuated Tlr4/Myd88/Nfκb signaling; however, the causal involvement of this pathway requires further validation.

This study has some limitations that must be noted here. The FMT and metabolite supplementation experiments in the present study were performed in mouse models rather than directly in suckling lambs. These experiments provided functional support for the associations observed in diarrheic suckling lambs, indicating that diarrhea-associated microbiota and the tryptophan-related metabolites IAld and 5-HIAA were associated with intestinal barrier function and inflammatory responses under controlled experimental conditions. However, owing to species differences and the experimental setting of the DSS model, the findings of this study should not be interpreted as direct evidence that diarrhea-associated microbiota can induce diarrhea-related microbial and metabolic phenotypes in lambs, nor as direct evidence that IAld and 5-HIAA exert therapeutic effects in suckling lambs. Future lamb-based FMT and IAld/5-HIAA supplementation studies are needed to determine whether diarrhea-associated microbiota can induce diarrhea-related microbial and metabolic phenotypes and whether these metabolites can improve intestinal barrier function and inflammatory status in suckling lambs. Such approaches will provide more direct evidence for microbiota-based interventions and antibiotic-alternative strategies in suckling lamb production.

## Conclusions

**Fig. 10:**
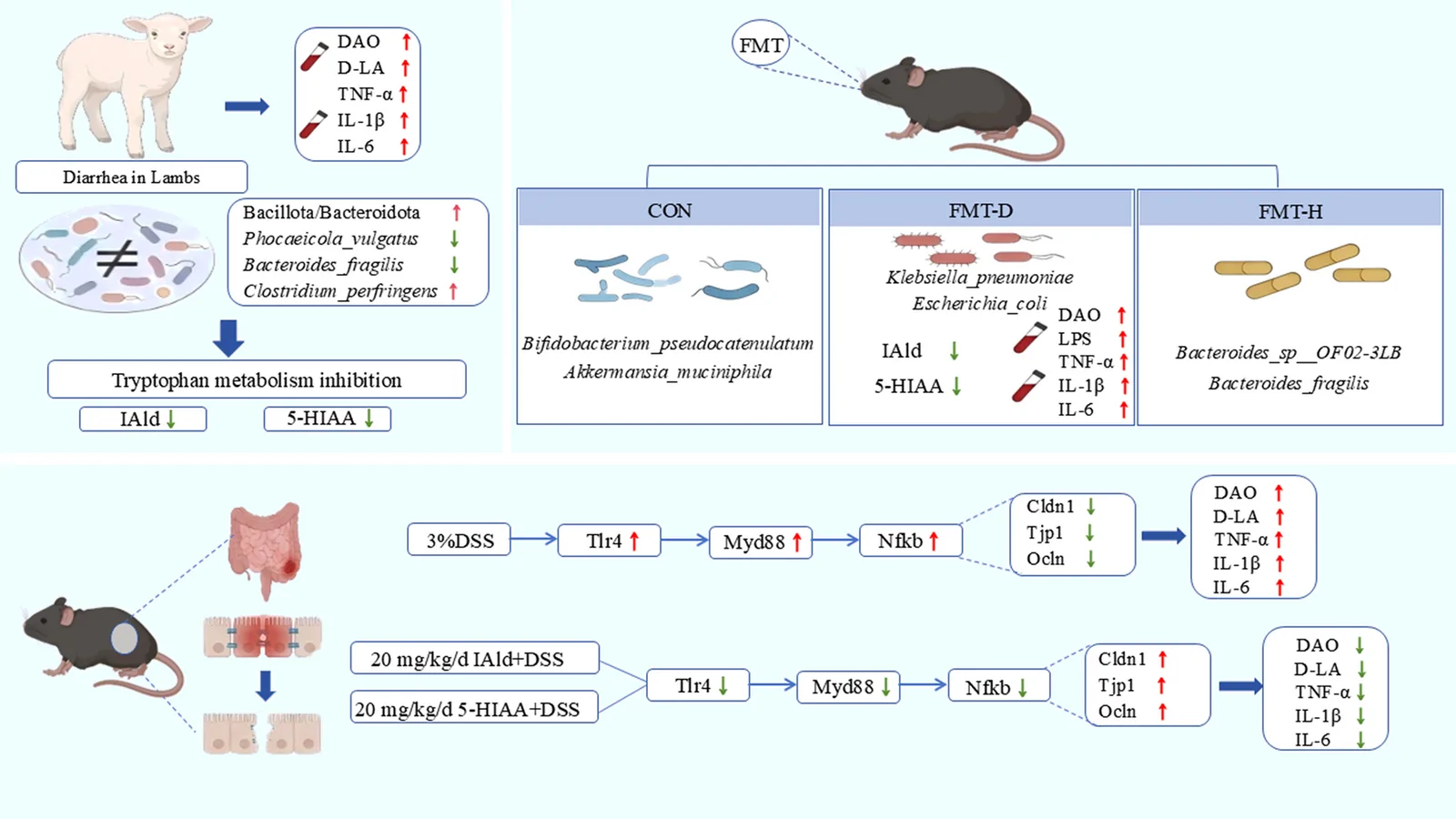
Reduced gut microbiota-derived indole-3-carboxaldehyde and 5-hydroxyindole-3-acetic acid are associated with intestinal barrier dysfunction and inflammation in diarrheic suckling lambs. The red-highlighted region indicates the distal colon affected by DSS-induced colitis

In summary, this study demonstrated that diarrhea in suckling lambs is closely associated with gut microbiota dysbiosis and disrupted tryptophan metabolism. This condition is characterized by elevated serum levels of DAO, D-LA, and the pro-inflammatory cytokines IL-6, TNF-α, and IL-1β, indicating increased intestinal permeability and an aggravated inflammatory response. Moreover, IAld and 5-HIAA alleviate inflammation and improve intestinal epithelial barrier function by enhancing tight junction integrity and reduced the expression of Tlr4/Myd88/Nfκb pathway-related inflammatory signaling molecules, thereby representing potential antibiotic alternatives for the prevention and control of diarrhea in healthy livestock production (Fig. 10).

## Supplementary Information

Additional file 1: Table S1. Sampling information of lambs used in the current study. Table S2. DAI scoring criteria. Table S3. Primers sequence for qRT-PCR. Table S4. Non-significant serum biochemical, antioxidant, anti-inflammatory, and immunoglobulin parameters in healthy and diarrheal lambs. Table S5. Core microbiome analysis. Fig. S1. The structure and composition of fecal microbiota in healthy and diarrheal suckling lambs. Fig. S2. Metabolomic analysis of fecal samples from healthy and diarrhea-affected suckling lambs. Fig. S3. FMT induced remodeling of the intestinal microbiota in germ free mice.

## Data Availability

The data used or analysed during the current study are available from the corresponding author on reasonable request.

## Abbreviations

5-HIAA: 5-Hydroxyindole-3-acetic acid
Cldn1: Claudin-1
DAO: Diamine oxidase
D-LA: D-Lactate
GSH-Px: Glutathione peroxidase
IgA: Immunoglobulin A
IgG: Immunoglobulin G
IgM: Immunoglobulin M
IAld: Indole-3-carboxaldehyde
IL-10: Interleukin-10
IL-1β: Interleukin-1β
IL-22: Interleukin-22
IL-6: Interleukin-6
LPS: Lipopolysaccharide
MDA: Malondialdehyde
Ocln: Occludin
ROS: Reactive oxygen species
SOD: Superoxide dismutase
T-AOC: Total antioxidant capacity
TNF-α: Tumor necrosis factor-α
Tjp1: Tight junction protein 1
