# Supplementary material for: Reduced gut microbiota-derived indole-3-carboxaldehyde and 5-hydroxyindole-3-acetic acid are associated with intestinal barrier dysfunction and inflammation in diarrheic suckling lambs
Source: J Anim Sci Biotechnol. 2026 Sep 4;17:169. doi: 10.1186/s40104-026-01493-w (PMC13543504; doi:10.1186/s40104-026-01493-w)
Supplement: Supplementary file 1 [file 40104_2026_1493_MOESM1_ESM.docx]

| **Table S1** Sampling information of lambs used in the current study | | | | | | | | | | |
| --- | --- | --- | --- | --- | --- | --- | --- | --- | --- | --- |
| **Lambs ID** | **Group** | **Sex** | **Age of sample collection, d** | **Age of first diarrea case, d** | **Fecal score** | **Coronavirus** | **Rotavirus** | **Cryptosporidium** | ***C. perfringens*** | **E. coli k99** |
| 3-4 | D_1 | male | 11 | 10 | 2 | - | - | - | + | + |
| 3-123 | D_2 | male | 10 | 9 | 3 | + | - | + | - | + |
| 4-153 | D_3 | male | 11 | 10 | 3 | - | - | + | - | + |
| 4-162 | D_4 | male | 12 | 11 | 3 | + | - | + | - | + |
| 3-186 | D_5 | male | 10 | 9 | 3 | + | - | - | + | - |
| 3-191 | D_6 | male | 10 | 9 | 3 | - | - | + | + | + |
| 4-189 | D_7 | male | 11 | 10 | 2 | + | - | - | + | - |
| 4-195 | D_8 | male | 11 | 10 | 3 | - | - | - | + | + |
| 3-206 | D_9 | male | 11 | 10 | 3 | + | - | + | + | - |
| 4-207 | D_10 | male | 12 | 11 | 2 | + | - | + | - | + |
| 3-209 | D_11 | male | 11 | 10 | 3 | + | - | + | + | + |
| 4-226 | D_12 | male | 12 | 11 | 2 | + | - | - | + | - |
| 4-176 | H_1 | male | 11 | / | 1 | - | - | - | - | - |
| 4-177 | H_2 | male | 10 | / | 0 | - | - | - | - | - |
| 3-164 | H_3 | male | 11 | / | 0 | - | - | - | - | - |
| 3-180 | H_4 | male | 10 | / | 0 | - | - | - | - | - |
| 4-183 | H_5 | male | 10 | / | 1 | - | - | - | - | - |
| 3-187 | H_6 | male | 11 | / | 0 | - | - | - | - | - |
| 4-184 | H_7 | male | 12 | / | 0 | - | - | - | - | - |
| 3-196 | H_8 | male | 12 | / | 0 | - | - | - | - | - |
| 3-202 | H_9 | male | 11 | / | 1 | - | - | - | - | - |
| 3-204 | H_10 | male | 11 | / | 0 | - | - | - | - | - |
| 4-216 | H_11 | male | 10 | / | 1 | - | - | - | - | - |
| 4-214 | H_12 | male | 10 | / | 1 | - | - | - | - | - |

**Table S2** DAI scoring criteria

| **Score** | **Weight loss %** | **Stool consistency** | **Blood in stool** |
| --- | --- | --- | --- |
| 0 | 0 | Normal | Normal |
| 1 | 1-5 | Soft | Normal |
| 2 | 5-10 | Fluid | Occult blood |
| 3 | 10-20 | Fluid | Occult blood |
| 4 | ≥20 | Diarrhea | Gross bleeding |

**Table S3** Primers sequence for qRT-PCR

| **Gene name** | **Forward (5′-3′)** | **Reverse (5′-3′)** |
| --- | --- | --- |
| *β-actin* | CCTAGGCACCAGGGTGTGAT | GGTTGGCCTTAGGGTTCAGG |
| *Cldn1* | GGGGACAACATCGTGACCG | AGGAGTCGAAGACTTTGCACT |
| *Tjp1* | GCCGCTAAGAGCACAGCAA | TCCCCACTCTGAAAATGAGGA |
| *Ocln* | CAGACCTGATGAATTCAAACCCA | AGAGTACGCTGGCTGAGAGA |
| *Nfκb* | ACACTGGAAGCACGGATGAC | TGTCTGTGAGTTGCCGGTCT |
| *Tlr4* | AGTGGTCAGTGTGATTGTGG | GATGCTTTCTCCTCTGCTGT |
| *Myd88* | GTAAGCAGCAGAACCAGGAGT | GCAGATAAAGGCATCGAAAAG |

**Table S4** Non-significant serum biochemical, antioxidant, anti-inflammatory, and immunoglobulin parameters in healthy and diarrheal lambs

| **Items** | **Health** | **Diarrhea** | ***P*-value** |
| --- | --- | --- | --- |
| LPS, EU/L | 1051.78±31.99 | 1098.58±33.37 | 0.322 |
| T-AOC, mmol/L | 1.10±0.02 | 1.10±0.32 | 0.965 |
| SOD, U/L | 147.96±4.64 | 145.10±3.15 | 0.615 |
| GSH-Px, U/mL | 79.22±4.14 | 78.28±3.42 | 0.863 |
| IL-10, pg/mL | 45.44±2.20 | 44.34±1.90 | 0.709 |
| IgG, g/L | 41.16±1.78 | 36.13±2.05 | 0.078 |
| IgA, g/L | 15.18±0.33 | 14.33±0.44 | 0.138 |
| IgM, g/L | 26.10±0.97 | 24.41±0.73 | 0.178 |

Statistical significance was calculated using Student’s unpaired *t*-test

Data are expressed as the mean ± SEM (*n* = 12)

**Table S5** Core microbiome analysis

| **Items** | **Abundance** | | | **Number** | | |
| --- | --- | --- | --- | --- | --- | --- |
|  | **Transient** | **Intermittent** | **Persistent** | **Transient** | **Intermittent** | **Persistent** |
| Lamb | | | | | | |
| Health | 0.85% | 18.27% | 80.87% | 5.69% | 68.36% | 25.95% |
| Diarrhea | 0.49% | 9.99% | 89.52% | 5.15% | 59.90% | 34.94% |
| Mouse | | | | | | |
| CON | 0.49% | 22.59% | 76.92% | 3.34% | 65.24% | 31.42% |
| FMT_H | 1.00% | 32.81% | 66.19% | 4.30% | 65.71% | 29.99% |
| FMT_D | 0.91% | 16.89% | 82.20% | 3.55% | 58.61% | 37.84% |

**Fig. S1** The structure and composition of fecal microbiota in healthy and diarrheal suckling lambs. **a** Shannon index represents the α-diversity. **b** Simpson index represents the α-diversity. **c** Linear discriminant analysis effect size

**Fig. S2** Metabolomic analysis of fecal samples from healthy and diarrhea-affected suckling lambs. **a** Partial least squares discriminant analysis (PLS-DA). **b** Venn analysis. **c** Metabolite classification pie charts

**Fig. S3** FMT induced remodeling of the intestinal microbiota in germ free mice. **a** ACE index represents the α-diversity. **b** Shannon index represents the α-diversity. **c** Based on the Kruskal-Wallis rank-sum test for significant differences at the phylum level. **d** Based on the Kruskal-Wallis rank-sum test for significant differences at the genus level. **e** Linear discriminant analysis effect size
